# Human Leukocyte Antigens and HIV Type 1 Viral Load in Early and Chronic Infection: Predominance of Evolving Relationships

**DOI:** 10.1371/journal.pone.0009629

**Published:** 2010-03-10

**Authors:** Jianming Tang, Rakhi Malhotra, Wei Song, Ilene Brill, Liangyuan Hu, Paul K. Farmer, Joseph Mulenga, Susan Allen, Eric Hunter, Richard A. Kaslow

**Affiliations:** 1 Department of Medicine, University of Alabama at Birmingham, Birmingham, Alabama, United States of America; 2 Department of Epidemiology, University of Alabama at Birmingham, Birmingham, Alabama, United States of America; 3 Vaccine Research Center, Emory University, Atlanta, Georgia, United States of America; 4 Rwanda-Zambia HIV-1 Research Group, Lusaka, Zambia; 5 Department of Pathology and Laboratory Medicine, Emory University, Atlanta, Georgia, United States of America; University of California San Francisco, United States of America

## Abstract

**Background:**

During untreated, chronic HIV-1 infection, plasma viral load (VL) is a relatively stable quantitative trait that has clinical and epidemiological implications. Immunogenetic research has established various human genetic factors, especially human leukocyte antigen (HLA) variants, as independent determinants of VL set-point.

**Methodology/Principal Findings:**

To identify and clarify HLA alleles that are associated with either transient or durable immune control of HIV-1 infection, we evaluated the relationships of HLA class I and class II alleles with VL among 563 seroprevalent Zambians (SPs) who were seropositive at enrollment and 221 seroconverters (SCs) who became seropositive during quarterly follow-up visits. After statistical adjustments for non-genetic factors (sex and age), two unfavorable alleles (A*3601 and DRB1*0102) were independently associated with high VL in SPs (*p*<0.01) but not in SCs. In contrast, favorable HLA variants, mainly A*74, B*13, B*57 (or Cw*18), and one *HLA-A* and *HLA-C* combination (A*30+Cw*03), dominated in SCs; their independent associations with low VL were reflected in regression beta estimates that ranged from −0.47±0.23 to −0.92±0.32 log_10_ in SCs (*p*<0.05). Except for Cw*18, all favorable variants had diminishing or vanishing association with VL in SPs (*p*≤0.86).

**Conclusions/Significance:**

Overall, each of the three HLA class I genes had at least one allele that might contribute to effective immune control, especially during the early course of HIV-1 infection. These observations can provide a useful framework for ongoing analyses of viral mutations induced by protective immune responses.

## Introduction

Polymorphic human leukocyte antigen (HLA) molecules facilitate immune surveillance by presenting a wide spectrum of self and foreign antigens to T-cells and by serving as ligands for killer immunoglobulin-like receptors (KIRs) on natural killer (NK) cells. The extensive HLA allelic diversity [1], [2], [3], [4] reflects positive, negative, and balancing selections by myriad human pathogens [5]. In the context of human immunodeficiency virus type 1 (HIV-1) infection, multiple HLA class I alleles have been shown to differentially influence viral pathogenesis, often as a result of their selective targeting of viral epitopes for cytotoxic T-lymphocyte (CTL) responses [6], [7], [8]. Such CTL responses frequently and often rapidly induce viral immune escape, regardless of patient populations or HIV-1 subtypes (clades) [8], [9], [10], [11], [12], [13], [14]. Viral adaptation to HLA class I-restricted, protective CTL responses can even reach fixation in a given population if the resulting viral mutations have little or no impact on viral fitness [15]. In contrast, HIV-1 variants with CTL-driven mutations that are associated with substantial fitness costs can readily revert to the wild-type once they inhabit individuals who lack the CTL-inducing HLA alleles or their equivalents [11], [16]. Understanding such intrinsic virus-HLA interplay at both the individual and population levels should help elucidate correlates of protection against HIV-1 infection, which are critical to the design of effective interventions.

Differential impact of HLA alleles on HIV-1 viral load (VL) was first observed among seroconverted men in the Multicenter AIDS Cohort Study [17], shortly after viral load was recognized as a clinically important outcome [18], [19]. More recently, at least three independent genome-wide association studies have confirmed that HLA genes are true quantitative trait loci (QTL) related to HIV-1 viremia [20], [21], [22]. These findings are consistent with other documented associations of HLA alleles or their supertypes with rates of HIV-1 disease progression (time to AIDS or CD4^+^ T-cell depletion) [23], [24], [25], [26], [27], although only a few HLA alleles have been considered as universally favorable or unfavorable.

In our own work, several HLA alleles and haplotypes have been associated with low (favorable) or high (unfavorable) VL in adult Zambians predominantly infected with HIV-1 clade C (HIV-1C) viruses [28]. HLA-B*57 as a universally favorable HLA variant [29] was further associated with reduced rate (and incidence) of HIV-1 transmission from seropositive Zambians to their cohabiting partners [30]. To refine and expand these observations in our enlarged Zambian cohort, we have evaluated the potentially distinctive HLA relationships in seroconverters (SCs) and seroprevalent patients (SPs).

## Results

### Overall Characteristics of HIV-1 Seropositive Zambians

**Figure 1:**
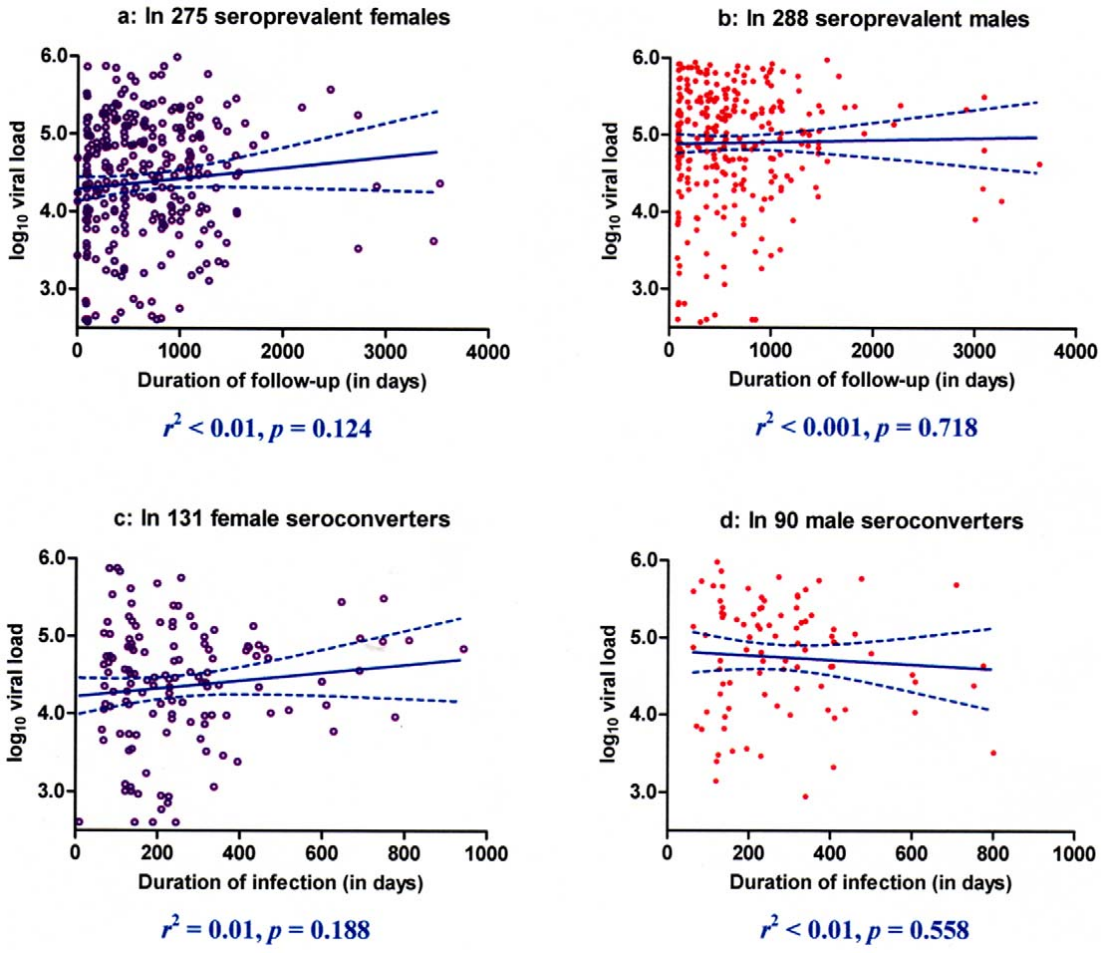
Distribution of HIV-1 viral load (VL) in the study population, with stratification by sex and serostatus at enrollment. There is no linear correlation between duration of follow-up and log_10_ VL in seroprevalent Zambians (*p*≥0.12) or duration of infection and log_10_ VL in seroconverters (*p*≥0.56). In each panel, the projected slope and its 95% confidence interval are shown in solid and dotted lines, respectively.

For this study, 784 HIV-1 seropositive Zambian adults consisted of 563 SPs who were seropositive at enrollment and 221 SCs who became seropositive during quarterly follow-up visits (**Table 1**). Most of them had the first known seropositive tests between 1996 and 2004. At the time of VL measurements, SPs had a median duration of follow-up (DOF) of 546 days, while SCs had a median duration of infection (DOI) of 229 days. DOF was defined as the time interval from enrollment to first plasma sample used for VL. DOI was the interval from the estimated time of HIV-1 infection (the midpoint between last seronegative test and first seropositive test) to the first plasma taken at least 63 days after infection. Except for a few outliers, DOF and DOI relative to VL measurements were <2,000 and <600 days, respectively (**Figure 1**). Neither DOF nor DOI correlated with log_10_ VL (*p*≥0.12). Between the two patient groups, statistically significant (*p*≤0.05) differences were seen for sex ratio, age, and the distribution of three VL categories previously shown to be highly predictive of viral transmission potential [30], [31], [32]. The minor difference between SPs and SCs in log_10_ VL was well within the boundary (0.30 log_10_) of biological and epidemiological relevance [19], [33]. For consistency with earlier work [28], sex and age group (≥40 versus <40 years) were retained as covariates (non-genetic factors) in subsequent association models.

**Table 1:** Characteristics of 563 Zambians who were HIV-1 seropositive at enrollment and 221 seroconverters who became seropositive during quarterly follow-up visits.

Characteristics	HIV-1 seroprevalents (SPs)	Seroconverters (SCs)	*p*
Dates of seropositive tests	08/1999 (10/1996-03/2004)	10/2001 (03/1998-03/2004)	NA
DOF or DOI (in days)	546 (252–962)	229 (134–334)	<0.0001
Sex ratio: M/F	288/275 (1.05)	90/131 (0.69)	0.009
Age (yrs, mean ± SD)	31.0±7.8	28.8±7.4	<0.001
Age group (n, %)			0.064
≥40 yrs	108 (19.2)	30 (13.6)	
<40 yrs	455 (80.8)	191 (86.4)	
Viral load categories (n, %)			0.034
Low (<10^4^ copies/mL)	105 (18.7)	51 (23.1)	
Medium (10^4^–10^5^)	232 (41.2)	103 (46.6)	
High (>10^5^ copies/mL)	226 (40.1)	67 (30.3)	
log_10_ viral load (mean ± SD)	4.71±0.78	4.54±0.77	0.006

Dates (month/year) of seropositive tests, duration of follow-up (DOF, for SPs), and duration of infection (DOI, for SCs) are shown as median and interquartile range (in parentheses). The difference between patient groups is assessed using Mann-Whitney U test. All other comparisons between patient groups are based on Student's *t*-test (for quantitative measures) and χ^2^ test (for categorical measures). F  =  female; M  =  male; NA  =  not applicable; SD  =  standard deviation of the mean.

### HLA Class I Alleles and Their Common Haplotypes in Zambians

For the 784 HIV-1 seropositive Zambians, molecular genotyping for the three HLA class I genes (*HLA-A*, *HLA-B*, and *HLA-C*) resolved most alleles to their 4-digit (high resolution) designations while leaving some at their 2-digit (allele group) specificities. HLA class I allele groups with multiple common alleles were limited to A*02, A*30, A*68, B*14, B*15, B*42, B*44, B*57, B*58, Cw*02, Cw*03, and Cw*07. The overall frequencies of HLA class I alleles and haplotypes (local and extended) in the study population were highly comparable to those seen in 429 discordant couples (858 individuals, including 204 who remained HIV-1 seronegative) analyzed previously for heterosexual HIV-1 transmission [30]. One striking observation in our population was the high frequency (17%) of B*1503-Cw*0210 (or B*15-Cw*02), a haplotype rarely reported elsewhere [34], [35], [36]. Eight HLA class I alleles (A*11, A*32, B*27, B*3502, B*3503, B*55, B*56, and Cw*05) reportedly associated with HIV-1 in populations of European ancestry [17], [22], [24], [37] were too rare to be assessed in Zambians (number of subjects ranged from one to 13).

Tests of linkage disequilibrium (LD) among HLA class I alleles focused on common allele groups seen in at least 16 individuals (∼2% of the study population). A total of 32 common, 2-locus haplotypes were identified (**Table S1**). Correlation coefficients (*r*) in pairwise LD tests ranged from 0.17 to 0.97 (*p*<0.001 for all). Only two pairs of alleles, B*39 with Cw*12 plus B*42 with Cw*17, were considered as tagging for each other (*r* ^2^>0.75, *p*<1×10^−16^). Accordingly, the vast majority of individual HLA class I alleles required independent testing in association analyses. Three common HLA class I combinations, A*23+B*14, A*23+Cw*07, and A*30+Cw*03, were also tested selectively because they were considered as probable haplotypes associated with HIV-1 VL in Zambians [28].

### HLA Class II Alleles and Their Common Haplotypes in Zambians

Alleles for two HLA class II genes, *HLA-DRB1* and *HLA-DQB1*, were fully resolved to their 4-digit designations in all but three of the 784 Zambians. Within the study population, 25 class II alleles found in at least 16 individuals qualified for formal association analyses. Strong linkage disequilibrium (LD) among alleles at these two neighboring class II loci allowed unambiguous assignment of 2-locus haplotypes in these subjects (probabilities >95% for all), leading to the identification of 19 common haplotyes (*r* = 0.22−0.95, *p*≤0.01) (**Table S1**). Only the neighboring alleles DRB1*0302 and DQB1*0402 were in sufficient LD to tag each other (*r* ^2^ = 0.89, *p*<1×10^−16^). One common haplotype, DRB1*1301-DQB1*0501, appeared to be unique to the Zambian population. The frequency of the haplotype DRB1*1101-DQB1*0602 exceeded that of DRB1*1101-DQB1*0301 in Zambians; that excess has not been reported in other populations of African ancestry [38]. These overall allele and haplotype frequency patterns were similar to those seen in 584 Zambians (including 151 HIV-1 seronegatives) analyzed earlier for heterosexual HIV-1 transmission [32].

### Analyses Using Categorical and Quantitative VL Measures

We used log_10_ VL and two contrasting VL categories (high versus low) as alternative outcome measures in generalized linear models (GLMs) and logistic regression models to screen the effects of common HLA alleles and haplotypes, including fully resolved HLA class I alleles (e.g., A*6801, A*6802, B*3501, B*1302, B*3910, B*5801, and B*5802) implicated in earlier studies of native Africans or African-Americans [28], [39], [40], [41], [42]. The VL measure was first evaluated in SPs and SCs combined to improve statistical power (sample size) [28], [35]. The comparison between the more extreme (categorical) VL outcomes excluded 335 patients (42.7%) with intermediate VL in order to reduce the potential misclassification (overlapping) between patient groups [26], [43].

In univariate GLMs, eight individual HLA variants and two *HLA-A* and *HLA-C* allele combinations were significantly associated with HIV-1 VL in this study population (*p*≤0.04) (**Table 2**). Five of these variants were favorable and five unfavorable, with the number of individuals carrying them ranging from 42 (5.4%) to 116 (14.8%). All variants from class I loci have shown similar associations elsewhere [28], [35]. B*57 and Cw*18, which were in strong LD (*r* = 0.75, *p*<1×10^−16^), were the most favorable alleles (mean β estimates ≤−0.34 log_10_); A*36 and DRB1*0102 were most unfavorable (mean β close to 0.30 log_10_ for both). These VL differences approached and usually exceeded the quantitative threshold (±0.30 log_10_, or ∼2-fold difference) considered biologically and epidemiologically significant [19], [33]. The weak association for Cw*16 (*p* = 0.08) was explained by its LD (*r* = 0.57, *p*<1×10^−16^) with an established unfavorable allele B*45 (*p* = 0.05).

**Table 2:** List of 11 HLA variants associated with HIV-1 viral load (VL) based on either generalized linear models (Series 1) or logistic regression analyses (Series 2).

	Series 1: all 784 Zambians			Series 2: high versus low VL		
HLA variants	No. of subjects	Mean β ± SE^c^	*p*	No. of subjects	OR (95% CI)^d^	*p*
A*36	95	**0.26±0.08**	0.001	54	**3.61 (1.66–7.88)**	0.001
A*74	108	**−0.21±0.08**	0.006	61	**0.54 (0.30–0.97)**	0.038
B*45^e^	106	**0.16±0.08**	0.043	65	1.62 (0.86–3.04)	0.133
B*57	93	**−0.34±0.08**	<0.0001	55	**0.31 (0.17–0.58)**	<0.001
B*5802	65	**0.21**±**0.10**	0.031	43	2.01 (0.92–4.38)	0.080
B*81	52	**−0.30**±**0.11**	0.005	35	**0.42 (0.20–0.89)**	0.024
Cw*16^e^	100	0.14±0.08	0.079	66	1.66 (0.88**–**3.14)	0.121
Cw*18	111	**−0.37**±**0.07**	<0.0001	67	**0.26 (0.15–0.47)**	<0.0001
DRB1*0102	63	**0.28±0.10**	0.005	38	**3.24 (1.34–7.86)**	0.009
A*23+Cw*07	56	**0.22**±**0.10**	0.033	33	2.09 (0.85–5.17)	0.111
A*30+Cw*03	42	−**0.33±0.12**	0.005	22	**0.34 (0.13–0.88)**	0.025

Association analyses begin with a typical, cross-sectional study design to emphasize statistical power (Series 1). In Series 2, subjects with medium VL (10^4^–10^5^ copies/mL) are excluded, with the assumption that they may occasionally obscure the classification of patients with low and high VL (Table 1). For consistency, sex and age are treated as covariates in each univariate analysis; statistically significant relationships (*p*<0.05) are shown in **bold**. CI  =  confidence interval; OR  =  odds ratio; SE  =  standard error.

Despite almost 43% reduction in the total sample size, the logistic regression models provided confirmatory findings for seven of 10 HLA variants detected in GLMs (**Table 2**). The associations of B*45, B*5802, and A*23+Cw*07 were no longer nominally statistically significant (*p* = 0.13, 0.08, and 0.11, respectively). Among the seven associations supported by logistic regression models, A*36 and DRB1*0102 were among the most unfavorable, while Cw*18 and B*57 were among the most favorable. These seven associations were further confirmed in alternative GLMs, where the parameter estimates improved for all (**Table S2**). For all 10 HLA variants associated with VL using either outcome, their rankings based on respective beta estimates and on odds ratios had a strong correlation (Spearman ρ = 0.99, *p*<0.0001). However, the same analytic approach failed to confirm the associations of 16 HLA variants with HIV-1 disease control, as previously reported for populations of European and African ancestry (**Table S3**).

### Stratified Analysis of SPs and SCs

For separate analyses of SPs and SCs, we first assessed the relationships across three categories of HIV-1 VL. Three alleles (A*36, A*74, and B*57) showed highly consistent effects on VL in both SPs and SCs (*p*≤0.02 in univariate Cochran-Armitage trend test in each patient group) (**Table 3**). Three associations (B*5802, Cw*18, and DRB1*0102) were restricted to SPs (*p* = 0.02, <0.0001, and <0.01, respectively). Modest LD between A*29 and B*13 (*r* = 0.27) (**Table S1**) appeared to account for the association of A*29 in SCs (*p* = 0.05): in the absence of B*13, the association of A*29 was no longer statistically significant (*p* = 0.09).

**Table 3:** Distribution of 14 HLA variants in HIV-1 seroprevalent and seroconverted Zambians stratified by three levels of plasma viral load.

	Distribution (n, %) in 563 seroprevalent Zambians (SPs)				Distribution (n, %) in 221 seroconverters (SCs)			
HLA variant	<10^4^ (*N* = 105)	10^4^–10^5^ (*N* = 232)	>10^5^ (*N* = 226)	*P*_trend_	<10^4^ (*N* = 54)	10^4^–10^5^ (*N* = 103)	>10^5^ (*N* = 67)	*P*_trend_
A*29	13 (12.4)	38 (16.4)	34 (15.0)	0.662	11 (21.6)	9 (8.7)	6 (9.0)	0.048
A*36	7 (6.7)	25 (10.8)	31 (13.7)	0.057	2 (3.9)	16 (15.5)	14 (20.9)	0.011
A*74	21 (20.0)	37 (16.0)	28 (12.4)	0.068	9 (17.7)	10 (9.7)	3 (4.5)	0.019
B*13	3 (2.9)	11 (4.7)	8 (3.5)	0.936	4 (7.8)	0	2 (3.0)	0.166
B*42	23 (21.9)	52 (22.4)	49 (21.7)	0.928	14 (27.5)	11 (10.7)	10 (14.9)	0.098
B*45	9 (8.6)	25 (10.8)	34 (15.0)	0.068	8 (15.7)	16 (15.5)	14 (20.9)	0.426
B*57	26 (24.8)	27 (11.6)	22 (9.7)	<0.001	7 (13.7)	11 (10.7)	0	0.005
B*5802	5 (4.8)	8 (3.5)	23 (10.2)	0.016	5 (9.8)	14 (13.6)	10 (14.9)	0.427
B*8101	11 (10.5)	16 (6.9)	12 (5.3)	0.096	6 (11.8)	1 (1.0)	6 (9.0)	0.711
Cw*16	10 (9.5)	21 (9.1)	35 (15.5)	0.054	6 (11.8)	13 (12.6)	15 (22.4)	0.094
Cw*18	31 (29.5)	36 (15.5)	23 (10.2)	<0.0001	8 (15.7)	8 (7.8)	5 (7.5)	0.154
DRB1*0102	2 (1.9)	14 (6.0)	23 (10.2)	0.004	5 (9.8)	8 (7.8)	8 (11.9)	0.641
A*23+Cw*07	5 (4.8)	15 (6.5)	21 (9.3)	0.113	2 (3.9)	8 (7.8)	5 (7.5)	0.481
A*30+Cw*03	7 (6.7)	15 (6.5)	7 (3.1)	0.106	6 (11.8)	5 (4.9)	2 (3.0)	0.052

HLA variants shown here have been confirmed in further analyses (generalized linear models, Table 4) or reported in earlier studies of native Africans with HIV-1C infection [28], [35], [41], [42].

Among HLA class I alleles associated with HIV-1 VL in either SPs or SCs or both groups, A*36 and B*5802 were unfavorable, while A*29, A*74, B*57, and Cw*18 were favorable; all of these were consistent with our earlier observations based on a smaller number of Zambians [28], [30], [41]. Alleles implicated in other studies (**Table S3**) [35], [41], [42] could not be confirmed in logistic regression models (**Table 3**) or alternative models (**Table S3**). Only B*57, represented primarily by B*5703 in Zambians, showed both strong internal consistency (between SPs and SCs) and unequivocal external corroboration [30], [44].

By GLM, including statistical adjustments for the effects of sex and age on HIV-1 VL, only B*57 (favorable) and B*5802 (unfavorable) were associated with log_10_ HIV-1 VL in both SPs and SCs (*p*≤0.05) (**Table 4**). Six additional associations were seen only in SPs: unfavorable for A*36, B*45, and DRB1*0102 (*p* = 0.004, 0.010, and 0.002, respectively) and favorable for B*8101, Cw*18, and A*30+Cw*03 (*p* = 0.025, <0.0001, and 0.034, respectively). In addition, A*29 and A*74 were favorable only in SCs (*p* = 0.015 and <0.001, respectively).

**Table 4:** Quantitative impact of 14 HLA variants on log_10_ HIV-1 viral load in two groups of Zambians.

	Tests in 563 seroprevalent Zambians (SPs)			Tests in 221 seroconverters (SCs)		
HLA variants	No. of subjects	β ± SE^b^	*p*	No. of subjects	β ± SE^b^	*p*
A*29	85	0.07±0.09	0.392	26	**−0.42**±**0.17**	0.015
A*36	63	**0.29±0.10**	0.004	32	0.21±0.16	0.189
A*74	86	−0.15±0.09	0.086	22	**−0.59±0.18**	<0.001
B*13	22	−0.03±0.16	0.860	6	**−0.78**±**0.33**	0.021
B*42	124	−0.01±0.08	0.905	35	−0.14±0.15	0.360
B*45	68	**0.25±0.09**	0.010	38	−0.04±0.15	0.804
B*57	75	**−0.28±0.09**	0.002	18	**−0.62±0.20**	0.002
B*5802	36	**0.28±0.13**	0.027	29	**0.33±0.16**	0.045
B*8101	39	**−0.27±0.12**	0.025	13	−0.29±0.23	0.204
Cw*16	66	0.16±0.10	0.096	34	0.02±0.15	0.886
Cw*18	90	**−0.39±0.08**	<0.0001	21	−0.30±0.19	0.113
DRB1*0102	39	**0.38±0.12**	0.002	21	−0.02±0.19	0.894
A*23+Cw*07	41	0.22±0.12	0.072	15	0.31±0.22	0.165
A*30+Cw*03	29	−**0.30±0.14**	0.034	13	−0.45±0.25	0.071

HLA variants are the same as those presented in Table 3. The parameter estimates (mean beta and standard error, SE) are statistically adjusted for sex and age; numbers in **bold** have adjusted *p* values≤0.05.

Biologically and epidemiologically meaningful differences (0.30 log_10_) in VL were observed for only three most favorable HLA alleles (A*29, A*74, and B*57) in SCs, their regression beta estimates ranged from −0.42±0.17 to −0.62±0.20 log_10_ (**Table 4**). However, in SPs the respective estimates diminished by at least 0.30 log_10_ (a two-fold difference). Although strong LD between B*57 and Cw*18 (*r* = 0.74 and 0.77 in SPs and SCs, respectively, *p*<1×10^−16^ for both) would have predicted similar impact of these two alleles in both patient groups, the impact of B*57 appeared stronger in SCs. B*13 (all B*1302 in this cohort) was clearly favorable in SCs (−0.78±0.33 log_10_, *p* = 0.02), but its effect vanished in SPs (0.03±0.16 log_10_, *p* = 0.86).

The effect sizes of the unfavorable A*36 and B*5802 varied little between SPs and SCs. For B*45 and DRB1*0102, the unfavorable effect observed in SPs was absent in SCs (**Table 4**). Again, the same analytic approach did not confirm or imply the involvement of other HLA alleles of major interest (**Table S3**) [[28], [35], [41], [42].

### Multivariable Analyses of HLA Variants and log_10_ VL

In a reduced multivariable model for SPs, A*36 and DRB1*0102 continued to show unfavorable association with VL (+0.25 and +0.38 log_10_, respectively, *p*<0.01 for both) (**Table 5**), while at least five HLA alleles and one HLA combination were retained as additional cofactors (adjusted *p*<0.05 for all) (**Table 5**). Based on their respective effect sizes (adjusted beta estimates), B*57, B*8101, and A*30+Cw*03 had similarly favorable impact on VL (∼−0.30 log_10_), and A*74 showed more modest impact (−0.20±0.08 log_10_). Cw*18 could replace B*57 and B*8101 as an independent factor (*p*<0.0001), but the association of B*57 and B*8101 was no longer statistically significant when the three variants were forced into the same model (results not shown).

**Table 5:** Multifactorial influences on log_10_ viral load (VL) in HIV-1 seroprevalent Zambians (SPs) and seroconverters (SCs).

	Reduced model 1: 563 SPs			Reduced model 2: 221 SCs		
Covariates	No. of subjects	β ± SE	*p*	No. of subjects	β ± SE	*p*
Sex = F	275	−0.53±0.06	<0.0001	131	−0.41±0.11	<0.001
Age ≥40 yrs	108	−0.08±0.08	0.344	30	0.36±0.15	0.020
A*36	63	0.25±0.10	0.009			
A*74	86	−0.20±0.08	0.017	22	−0.59±0.17	<0.001
B*13				6	−0.92±0.32	0.004
B*57	**75**	**−0.30±0.09**	**0.001**	**18**	**−0.60±0.19**	**0.002**
B*8101	39	−0.28±0.12	0.019			
Cw*18	**90**	**−0.39±0.08**	**<0.0001**	**21**	**−0.37±0.18**	**0.041**
A*30+Cw*03	29	−0.32±0.14	0.020	13	−0.47±0.23	0.045
DRB1*0102	39	0.38±0.12	0.002			

The parameter estimates (mean beta and standard error, SE) are adjusted for all factors retained in each model. Because of strong LD (see text), B*57 and Cw*18 (in **bold**) are entered into separate models for both SPs and SCs.

In SCs, favorable HLA class I alleles and one 2-allele combination dominated the independent effects, with mean beta estimates ranging from −0.47 to −0.92 log_10_ (∼3.0- to 8.3-fold) (*p*<0.05). These effects were ranked as B*13>B*57>A*74>(A*30+Cw*03) or B*13>A*74>Cw*18>(A*30+Cw*03) when B*57 was replaced Cw*18 in the reduced multivariable model. The adjusted effect sizes attributed to all five favorable HLA variants in aggregate exceeded the effect sizes for age and sex. In contrast to the model for SPs, a model for SCs that included both B*57 and Cw*18 indicated a non-significant association with Cw*18 (data not shown).

In further assessment of favorable and unfavorable HLA variants retained in the multivariable models (**Table 5**), it was evident that they did not segregate by a) specific *HLA-A* and *HLA-B* supertypes, including those that have been added or refined recently [45], b) HLA serological groups (properties as alloantigens), or c) relative frequency that might introduce allelic bias in homozygosity and heterozygosity within the study population.

## Discussion

Our expanded analyses of VL as a quantitative trait (log_10_ copy number) or as a categorical outcome measure in adult Zambians confirmed most of the favorable and unfavorable HLA associations that we previously reported [28]; this study also yielded three new, internally consistent findings. First, the associations of most of the favorable HLA class I alleles were more readily detected in seroconverters (SCs) than in seroprevalent subjects (SPs), even though SPs outnumbered SCs by 2-fold. HLA-A*74, B*57 (or Cw*18), and A*30+Cw*03 were clearly favorable; B*13 also appeared favorable in the few SCs with this allele. For each of these variants, its biologically and epidemiologically meaningful impact on VL either substantially diminished or totally vanished in SPs. Second, individual alleles and haplotypes at HLA class II loci were not prominently associated with altered viremia at the population level. Only one class II allele (DRB1*0102) was associated with high VL, and only in SPs. No other HLA class II allele or haplotype, including any previously reported to be associated with HIV-1 transmission or acquisition [[28], [35], [41], [42], showed appreciable impact on VL in either patient group. Third, an alternative study design that included only about 60% of the population with the two extreme categories of VL yielded very similar results (**Table S2**). In resource-limited settings that require concentration on patients with extreme outcomes [26], [43], considerable savings in cost may be achieved with relatively little loss of power, especially for exploratory studies of genetic determinants.

Plasma VL during untreated, chronic HIV-1 infection reflects an equilibrium between viral replication and immunologically mediated viral clearance. Population-based studies have clearly established the predictive value of VL for heterosexual HIV-1 transmission [31], [46] as well as time to AIDS, especially in Caucasian males [18], [47], [48]. Although our initial work indicated that variants from all three HLA class I genes were associated with VL in chronically infected Zambians [28], similar work based on a South African cohort has shown that *HLA-B* allele played the most dominant role [35]. In the same South African cohort, several favorable HLA alleles (B*42, B*57, B*5801, and B*8101) were further predictive of CD4^+^ T-cell slope over a 2-year follow-up period [42]. Our analyses here did not support the favorable association of B*42, although A*29 as a marginally favorable allele in Zambians (seroconverters only) showed weak LD with B*42. Two other *HLA-A* alleles (A*36 as unfavorable and A*74 as favorable) had even stronger associations, none of which could be explained by effects of their accompanying *HLA-B* and *HLA-C* alleles. Both alleles have been confirmed in recent analyses of African-Americans (unpublished results, available from RAK and JT). Thus, although the influences of multiple *HLA-B* variants on HIV-1 pathogenesis and evolution have been more readily detected and confirmed, several *HLA-A* alleles can exert comparable effects.

*HLA-B* and *HLA-C* genes are located in a genomic segment (the beta block) rarely disrupted by recombination hot spots [49], [50], [51], [52], [53]. As a result, the associations of two *HLA-C* alleles (Cw*16 as unfavorable and Cw*18 as favorable) with VL in Zambians could be partially or completely explained by their strong LD with *HLA-B* alleles: B*45 for Cw*16 and B*57 plus B*8101 for Cw*18. However, as we noted earlier [30], experimental evidence does suggest that Cw*1801 is able to present viral epitopes for cytotoxic T-lymphocyte (CTL) responses, which might account for its relatively durable impact on VL. Identification of one favorable A-C combination (A*30+Cw*03) further supported the role of *HLA-C* alleles in HIV-1 infection, because none of these alleles individually had a clear association with VL. Assuming that alleles at *HLA-C* and *HLA-A* can act in concert to confer genetic advantage during HIV-1 infection (or disadvantage in other settings), differences in peptide-binding and in *HLA-C* allelic expression can offer two plausible explanations [20], [54]. Of note, environmental factors can further complicate the genotype-phenotype relationships by influencing gene expression in leukocytes [55].

HLA class II molecules specialize in presenting exogenous antigens for immune surveillance by CD4^+^ T-helper (T_H_) cells. Their peptide-binding grooves can be loaded with protein degradation products (13–25 amino-acid residues) generated in the lysosome. CD4^+^ T-cells that effectively respond to HIV-1 antigens can facilitate immune control through cytokines and T-cell-dependent antibody responses, but activated T_H_ cells, especially those specific to HIV-1 antigens, are lost rapidly because they are preferentially targeted by the virus [56]. Striking a balance between the two functions can be difficult. Recent work has identified 33 HIV-1 epitopes recognized by CD4^+^ T-cells in patients with chronic HIV-1C infection [57]. Direct interactions between HIV-1 and HLA class II molecules have also been reported [58], [59], [60], [61]. Among individual HLA class II alleles, DRB1*1301 has been seen as a favorable allele in various populations [23], [32], [62]. Our analyses here failed to confirm any of the previously reported HLA class II alleles or haplotypes. Instead, DRB1*0102 was associated with higher VL, but only in SPs. The relationship counters to the hypothesized early involvement of class II molecules, before the precipitous loss of CD4^+^ T-cells. The largely inconclusive findings for HLA class II alleles and haplotypes might reflect their promiscuity in antigen presentation.

Within the context of HIV-1C infection, the Zambian cohort has produced some of the first evidence that HLA factors can mediate HIV-1 VL as well as heterosexual transmission [28], [30], [32]. Identification of favorable HLA factors in this cohort is particularly encouraging, as relatively few HLA alleles have unequivocal (consensus) effects in various investigations, especially in the era of highly active antiretroviral therapy [29]. For the few favorable HLA class I alleles confirmed in the Zambian, several have known or predicted HIV-1 epitopes [12], [63], [64]. For example, epitope-specific functional correlation is well documented for B*57 [44], [65]. B*13 (all B*1302 in Zambians) is another class I allele repeatedly observed as favorable [7], [42], [66], [67], [68], [69]. The impact of the favorable HLA class I alleles and haplotypes on VL was consistently more prominent in SCs than in SPs, suggesting that immune control mediated by favorable alleles like B*57 and B*13 diminish with time, most likely due to viral immune escape and accumulation of compensatory mutations [11], [12], [70], [71], [72]. Further association of favorable HLA alleles (B*57 and Cw*18) with reduced rates and incidences of heterosexual HIV-1 transmission in the Zambian cohort was also most apparent during the early follow-up period [30]. Continuing efforts to monitor the evolution of viral epitopes targeted by favorable HLA variants should provide critical guidance for HIV-1 vaccine design and clinical trials.

When expressed as a log_10_ value (a continuous variable), VL differences greater than 0.30 log_10_ (∼2-fold) are often considered biologically and epidemiologically significant [19], [33]. By our analyses, at least four favorable HLA class I alleles and one favorable combination independently conferred biologically and epidemiologically important impact on VL in SCs (**Table 5**). By contrast, the absence of advantageous class II variants highlights the importance of CTL and likely natural killer (NK) cell activities mediated by the three HLA class I genes. In particular, one or both of these pathways must be effective in controlling HIV-1 infection, since VL set-point is typically reached within the first nine weeks after HIV-1 infection [73], before the debut of high-affinity neutralizing antibody responses [74], [75]. Again, collection of longitudinal VL and viral sequencing data from SCs will help determine the timing and scope of viral immune escape, as already reported for HIV-1 *gag* variants [13], [44]. In addition, ongoing elucidation of KIR genotypes should set the stage for thorough, systematic evaluation of HLA-KIR interaction, which is the second and potentially critical pathway by which HLA class I alleles may regulate HIV-1 infection.

## Materials and Methods

### Ethics Statement

This study complied with the human experimentation guidelines of the United States Department of Health and Human Services, and all enrolled patients provided written informed consent. The work presented here was further approved by the Institutional Review Boards at University of Alabama at Birmingham, under Protocols X051108005 and X071119002.

### Study Population, HIV-1 Viral Load (VL), and HLA Genotyping

Beginning in 1995, HIV-1 seropositive Zambians were recruited and followed by the Rwanda/Zambia HIV-1 Research Group in Lusaka, Zambia. Procedures for quarterly medical examination, voluntary counseling and testing, and viral sequencing have been described elsewhere [31], [65], [76], [77], [78]. Measurements of plasma HIV-1 VL (RNA copies) were made in most cases with the Roche Amplicor 1.0 assay (Roche Diagnostics Systems Inc., Branchburg, NJ), which had a lower limit of detection (LLD) of 400 RNA copies per ml of plasma. HLA genotyping relied on a combination of PCR-based methods, including PCR with sequence-specific primers (SSP) (Dynal/Invitrogen, Brown Deer, WI), automated sequence-specific oligonucleotide (SSO) probe hybridization (Innogenetics, Alpharetta, GA), and sequencing-based typing (SBT) (Abbott Molecular, Inc., Des Plaines, IL) using capillary electrophoresis and the ABI 3130xl DNA Analyzer (Applied Biosystems, Foster City, CA) [30], [79]. HLA alleles were resolved to the first four digits, which correspond to distinct protein products designated by the World Health Organization Nomenclature Committee for Factors of the HLA System [80], [81].

### Computational Assignment of Local and Extended HLA Haplotypes

Using the expectation-maximization (EM) algorithm in SAS Genetics (SAS Institute, Cary, NC), common HLA class I B-C haplotypes were assigned for *HLA-B* and *HLA-C* alleles because of their known, strong linkage disequilibrium (LD) in the study population [30]. DRB1-DQB1 haplotypes were assigned for *HLA-DRB1* and *HLA-DQB1* alleles in the same way and for the same reason [32]. Assignments of these 2-locus (local) haplotypes helped to distinguish allelic from haplotypic effects. Assignments of extended haplotypes were assessed by relative LD (D') and correlation coefficients (*r*). In a given individual, simultaneous assignments of two haplotypes with a statistical probability ≥70% by the EM algorithm were considered reliable. Individuals with unreliable haplotype assignments (probabilities <70%) were excluded from formal testing of haplotypic associations.

### Descriptive Statistics

The overall characteristics of HIV-1 seroprevalent Zambians (SPs) and seroconverters (SCs) with complete HLA genotyping were summarized in **Table 1**. Differences between the two patient groups were assessed using Mann-Whitney U test (fro duration of infection or follow-up), Student's *t*-test (for age and log_10_ viral load), and χ^2^ test (for sex ratio, age group, and three categories of viral load).

### Analyses of HLA Variants in Relation to HIV-1 VL

The statistical approach to association analysis followed the strategies established in other related work [28], [30], [31], [82], [83], except that a) enlarged sample size (improved statistical power) facilitated separate analyses of 563 SPs versus 221 SCs, b) 56 individuals (32 SPs and 24 SCs) representing couples with unlinked viruses were retained in analyses because HLA genotyping had already been completed before non-identity of the virus in suspected recipients (seroconverters) was established; c) 19 SPs from cohabiting couples with short (<9 mth) follow-up were also included; d) 18 patients (15 SPs and 3 SCs) with VL less than the lower limit of detection (400 copies/mL) were excluded; e) several univariate models were presented in order to facilitate meta-analysis; f) software packages in SAS was updated to version 9.2 (SAS Institute), and g) results sorted in SAS were used to produce graphs using GraphPad Prism version 5.0 (http://www.graphpad.com/prism/Prism.htm). Summary statistics in association analyses included: 1) proportional odds ratio (pOR) and 95% confidence intervals (CIs); 2) regression beta estimates, expressed as means and standard errors (SE), and 3) univariate and multivariable (adjusted) *p* values. A *p* value≤0.050 was considered statistically significant because most HLA alleles and haplotypes highlighted in this work have been implicated in earlier studies. The overall emphasis was on biologically and epidemiologically significant (>0.30 log_10_) differences in VL [19], [33].

## Supporting Information

Table S1 HLA class I and class II alleles and haplotypes found in at least 16 out of 784 adult Zambians.
(0.04 MB DOC)

Table S2 HLA variants associated with log_10_ HIV-1 viral load (VL) based on alternative generalized linear models.
(0.05 MB DOC)

Table S3 Dismissal of 16 candidate HLA variants reported in earlier studies and common in Zambians.
(0.07 MB DOC)
