# Supplementary material for: Human Leukocyte Antigens and HIV Type 1 Viral Load in Early and Chronic Infection: Predominance of Evolving Relationships
Source: PLoS One. 2010 Mar 10;5(3):e9629. doi: 10.1371/journal.pone.0009629 (PMC2835758; doi:10.1371/journal.pone.0009629)
Supplement: Table S1 — HLA class I and class II alleles and haplotypes found in at least 16 out of 784 adult Zambians. (0.04 MB DOC) [file pone.0009629.s001.doc]

**Table S1.** HLA class I and class II alleles and haplotypes found in at least 16 out of 784 adult Zambians (sorted by allele names).
