# Supplementary material for: Human Leukocyte Antigens and HIV Type 1 Viral Load in Early and Chronic Infection: Predominance of Evolving Relationships
Source: PLoS One. 2010 Mar 10;5(3):e9629. doi: 10.1371/journal.pone.0009629 (PMC2835758; doi:10.1371/journal.pone.0009629)
Supplement: Table S2 — HLA variants associated with log10 HIV-1 viral load (VL) based on alternative generalized linear models. (0.05 MB DOC) [file pone.0009629.s002.doc]

**Table S2.** HLA variants and log_10_ HIV-1 viral load (VL) as tested in alternative, generalized linear models.

| HLA variants | Tests after excluding subjects with medium VL^a^ | | |
| --- | --- | --- | --- |
|  | No. of subjects | Mean beta ± SE^b^ | *p* |
| A*36 | 54 | **0.43 ± 0.13** | 0.001 |
| A*74 | 61 | **-0.28 ± 0.13** | 0.025 |
| B*45^c^ | 65 | 0.21 ± 0.12 | 0.092 |
| B*57 | 55 | **-0.46 ± 0.13** | <0.001 |
| B*5802 | 43 | 0.23 ± 0.15 | 0.111 |
| B*8101 | 35 | **-0.41 ±0.16** | 0.011 |
| Cw*16^c^ | 66 | 0.20 ± 0.12 | 0.106 |
| Cw*18 | 67 | **-0.53 ± 0.12** | <0.0001 |
| DRB1*0102^d^ | 38 | **0.38 ± 0.15** | 0.014 |
| A*23+Cw*07 | 33 | 0.24 ± 0.17 | 0.141 |
| A*30+Cw*03 | 22 | -**0.61 ± 0.20** | 0.003 |

^a^ Subjects with medium VL (10^4^-10^5^ copies/mL) are removed as they may occasionally obscure the classification of patients with low and high VL (as defined in Table 1).

^b^ For consistency, parameter estimates (beta and standard error, SE) are adjusted for sex and age; those with *p* <0.05 are shown in **bold**.

^c^ B*45 and Cw*16 are in strong linkage disequilibrium (see Table S1).

^d^ The allele is found exclusively on the DRB1*0102-DQB1*0501 haplotype, but DQB1*0501 alone has no association.
