# Supplementary material for: Human Leukocyte Antigens and HIV Type 1 Viral Load in Early and Chronic Infection: Predominance of Evolving Relationships
Source: PLoS One. 2010 Mar 10;5(3):e9629. doi: 10.1371/journal.pone.0009629 (PMC2835758; doi:10.1371/journal.pone.0009629)
Supplement: Table S3 — Dismissal of 16 candidate HLA variants reported in earlier studies and common in Zambians. (0.07 MB DOC) [file pone.0009629.s003.doc]

**Table S3.** Dismissal of 16 candidate HLA variants reported in earlier studies and common in Zambians.

| HLA variants^c^ | No. of  subjects | Logistic regression models^a^ | | GLMs for log_10_ VL^b^ | |
| --- | --- | --- | --- | --- | --- |
|  |  | pOR (95% CI)^c^ | *p* | Mean beta ± SE^d^ | *p* |
| A*02 | 169 | 0.93 (0.67-1.29) | 0.663 | -0.05 ± 0.06 | 0.411 |
| A*2301 | 190 | 1.26 (0.92-1.72) | 0.145 | 0.11 ± 0.06 | 0.068 |
| A*6802 | 161 | 1.29 (0.92-1.79) | 0.136 | 0.11 ± 0.07 | 0.087 |
| B*18 | 48 | 1.14 (0.65-1.98) | 0.653 | 0.09 ± 0.11 | 0.419 |
| B*3501 | 50 | 0.81 (0.47-1.39) | 0.443 | 0.00 ± 0.11 | 0.989 |
| B*39 (or B*39-Cw*12) | 19 | 0.54 (0.23-1.26) | 0.154 | -0.26 ± 0.17 | 0.124 |
| B*44 | 86 | 0.83 (0.55-1.28) | 0.401 | -0.11 ± 0.09 | 0.192 |
| B*5301 | 156 | 1.26 (0.90-1.76) | 0.180 | 0.08 ± 0.07 | 0.210 |
| B*5801 | 72 | 0.91 (0.58-1.44) | 0.685 | -0.05 ± 0.09 | 0.606 |
| A*02-Cw*16 | 42 | 1.09 (0.60-1.97) | 0.789 | 0.00 ± 0.12 | 0.983 |
| A*23+B*14 | 33 | 1.41 (0.72-2.76) | 0.312 | 0.21 ± 0.13 | 0.108 |
| DRB1*1301 | 120 | 0.78 (0.54-1.13) | 0.192 | -0.11 ± 0.07 | 0.119 |
| DRB1*1503 | 227 | 1.05 (0.78-1.40) | 0.764 | 0.06 ± 0.06 | 0.334 |
| B*14-Cw*08 | 103 | 0.95 (0.64-1.41) | 0.801 | -0.01 ± 0.08 | 0.865 |
| B*1503-Cw*0210 | 125 | 1.23 (0.86-1.78) | 0.260 | 0.10 ± 0.07 | 0.155 |
| DRB1*1302-DQB1*0609 | 37 | 0.90 (0.48-1.68) | 0.734 | -0.10 ± 0.13 | 0.436 |

^a^ Tests are based on 784 HIV-1 seropositive Zambians divided into three ordinal groups, i.e., high, medium, and low viral load (as defined in Table 1). Separate analyses of SPs and SCs confirm the lack of associations. CI = confidence interval; pOR = proportional odds ratio.

^b^ Sex and age are treated as cofactors in all generalized linear models (GLMs).

^c^ Additional variants like A*11, A*32, B*27, B*3502, B*3503, B*55, and Cw*05 can not be tested because of their rarity in the study population.
